# Are Risk-Taking and Ski Helmet Use Associated with an ACL Injury in Recreational Alpine Skiing?

**DOI:** 10.3390/ijerph16173107

**Published:** 2019-08-26

**Authors:** Gerhard Ruedl, Markus Posch, Martin Niedermeier, Klaus Greier, Martin Faulhaber, Alois Schranz, Martin Burtscher

**Affiliations:** 1 Department of Sport Science, University of Innsbruck, 6020 Innsbruck, Austria; 2 University College of Education (KPH) Stams, 6422 Stams, Austria; 3 Medalp Sportclinic, 6460 Imst, Austria

**Keywords:** alpine skiing, ACL injury, risk factor, risk-taking, risk compensation, ski helmet

## Abstract

According to the risk compensation hypothesis, the use of a ski helmet might provide a false sense of security, resulting in a riskier behavior by skiing faster or more aggressively, which might lead to an increased injury risk. Injury of the anterior cruciate ligament (ACL) is a common diagnosis in downhill skiers. Thus, the aim of the study was to evaluate the potential impact of risk-taking and ski helmet use on ACL injury risk in recreational skiing. Eighty-two ACL injured and 446 uninjured skiers with a mean age of 37.3 ± 11.9 years (52% females) were surveyed during the winter season 2018/19 about age, sex, self-reported risk-taking behavior, self-reported skill level, perceived speed, and ski helmet use. Multiple regression analysis revealed that older age (OR: 1.3, 95% CI: 1.2–1.4), riskier behavior (OR: 5.4, 95% CI: 2.8–10.5), and lower skill level (OR: 6.7, 95% CI: 3.4–13.3) were found to be factors associated with ACL injury, while ski helmet use was not. In conclusion, no support for the risk compensation hypothesis was found with regard to ACL injuries. Therefore, we doubt that ski helmet use increases the risk for ACL injury and recommend wearing a ski helmet due to reported protective effects.

## 1. Introduction

Recreational alpine skiing is one of the most popular winter sports annually enjoyed by several hundred million skiers worldwide [1]. In alpine skiing, the knee joint is the most common anatomical location of an injury with about one third of all injuries [2]. One of the most frequent diagnosis in injured recreational alpine skiers is a rupture of the anterior cruciate ligament (ACL) with 15–21% of all injuries [3,4]. In addition, up to 20% of all injuries on ski slopes are head injuries [1]. Head injury risk, however, can be significantly diminished by wearing a ski helmet [1,5]. Shealy et al. [5] found in a study from the winter seasons 1995/1996 to 2011/2012 a reduced incidence of any head injury and of potentially severe head injuries by 62% and by 67%, respectively, while helmet usage rate increased over the same time from 8 to 84%. However, the usage of a ski helmet might have additional, more subtle, effects on skiers.

According to the so-called risk compensation hypothesis, people adjust their behavior in accordance to their perceived level of risk [6,7]. Perceived level of risk might be diminished when wearing a ski helmet and might result in a riskier behavior on ski slopes by skiing faster or more aggressively or on more difficult runs [6,7]. Consequently, there is an ongoing debate whether the use of a ski helmet provides a false sense of security and leads to an increased injury risk of other body parts than the head.

Sulheim et al. [8] found among a cohort of 700 uninjured skiers that those who reported themselves as risk-takers were more likely to wear a helmet than skiers who viewed themselves as cautious skiers. In addition, other studies questioning uninjured skiers and snowboarders reported higher risk taking among helmet wearers [9,10,11]. 

In contrast, Scott et al. [7] tested the risk compensation hypothesis for safety helmets among a cohort of about 1800 uninjured skiers and snowboarders. These authors reported that users of helmets skied at self-perceived lower speeds and challenged themselves less than non-helmet users concluding that no evidence of risk compensation among helmet wearers in this study exists [7]. Ruedl et al. [12] measured mean speeds on ski slopes with a radar speed gun of more than 500 uninjured skiers and snowboarders and subsequently asked people about their self-rating of being a cautious or a risk-taking skier/snowboarder. Self-reported risk-taking behavior on ski slopes was associated with younger age, higher skiing ability, male sex, lower BMI, and with on average 8 km/h higher speed, but not with helmet use [12]. In a subsequent study, Ruedl et al. [13] found among a cohort of uninjured skiers and snowboarders that male sex, younger age, skiing, a higher skill level, a higher mean skiing time per season, and higher scores on the sensation seeking scale form V (SSS-V), but not helmet use, were predictive for a self-reported riskier behavior [13]. In addition, regarding risk compensation, 25% of helmet wearers believed that they ski/snowboard faster or in a more risky way by wearing a ski helmet and they showed a significantly higher score on the SSS-V compared to those helmet users not reporting risk compensation [13]. Willick et al. [14] found among a cohort of uninjured skiers and snowboarders that individuals reporting sometimes wearing a helmet scored significantly higher on the brief sensation seeking scale compared to those reporting never wearing a helmet or always wearing one whereas the latter two groups did not significantly differ from each other. In addition, risk compensation was significantly associated with sometimes wearing a helmet compared with always wearing a helmet [14].

If risk compensation due to ski helmet use would increase risk-taking and, therefore, also general injury risk on ski slopes, one would expect some differences among injured helmet wearers and non-wearers. However, Hagel et al. [6] found in a cohort of about 3300 injured skiers and snowboarders no evidence that helmet use increased the risk of severe injury or high-energy crash circumstances due to a higher speed. Their results suggest that helmet use in skiing and snowboarding is not associated with riskier activities that lead to non-head-neck injuries [6]. In addition, in a study by Ruedl et al. [15] including more than 2000 injured skiers and snowboarders, self-reported riskier behavior was associated with younger age, male sex, Austrian nationality, higher skill level, and off slope skiing and snowboarding, respectively, while ski helmet use and accident causes did not significantly differ between risky and cautious people. Thus, the authors concluded that with regard to helmet use, risk compensation was likely not present among injured recreational skiers and snowboarders [15]. 

Nevertheless, to evaluate the potential impact of risk-taking and ski helmet use on injury risk on ski slopes, a case-control study design including injured as well uninjured persons is necessary. To the best of our knowledge, up to now, factors associated with the risk compensation theory were investigated in either injured or uninjured recreational skiers/snowboarders only, but not in a sample of both injured cases and uninjured controls. However, if ski helmet use alters risk-taking behavior, one would expect an increased injury risk of other body parts than the head. Therefore, as an ACL injury is a common diagnosis in injured recreational alpine skiers [3,4], the primary aim of this study was to evaluate whether risk-taking behavior and ski helmet use are associated with an ACL injury in recreational skiers. A secondary aim was to evaluate potential factors associated with self-reported risk taking behavior.

## 2. Materials and Methods 

### 2.1. Design, Procedure, and Participants

This study was conducted as a case-control study of ACL-injured and uninjured female and male recreational alpine skiers during the winter season 2018/19 in a large Austrian ski area. This study has been approved by the Institutional Review board of the Department of Sport Science, Innsbruck and the ethical advisory board of the University of Innsbruck (18.07.2016). Cases and controls were informed about the aims of the study and gave their written informed consent for participating. 

Cases were interviewed in a ski clinic, which is directly located in the ski area, between December 2018 and April 2019 on 17 days using a questionnaire including questions on demographics as well as on potential intrinsic and extrinsic risk factors. ACL injury was diagnosed via magnetic resonance imaging (MRI). Inclusion criteria were a skiing-related noncontact ACL injury after a self-inflicted fall, an age >17 years and the use of any type of carving ski. Uninjured control participants were randomly selected at different spots in the same ski area mostly at the same days to minimize the potential impact of environmental factors (e.g., weather and slope conditions) on ACL injury risk [16]. The questionnaire on demographics and potential risk factors used for cases was also used for controls. Inclusion criteria were an age >17 years and the use of any type of carving ski.

### 2.2. Measurements

According to the questionnaire used in previous studies by Ruedl et al. [17] and Posch et al. [16] on ACL injuries among recreational skiers, cases and controls in this study were asked for age, sex, body weight, body height, self-reported risk-taking behavior (more risky versus more cautious), and self-reported skill level (classified in expert, advanced, intermediate, and beginner). Furthermore, participants were divided into more skilled (expert and advanced) and into less skilled (intermediate and beginner) skiers, as a tendency was shown to underestimate individual skiing skills, especially among female skiers [18]. In addition, cases had to rate their own perceived speed at the moment of injury (very fast, fast, moderate, slow, very slow) and controls were asked about their preferred skiing speed during the skiing day (very fast, fast, moderate, slow, very slow). Subsequently, due to a low number of skiers who perceived their skiing speed as very slow (*n* = 8) and very fast (*n* = 5), we decided to use only three different speed perception categories (slow/very slow, moderate, fast/very fast) according to Brunner et al. [19]. Finally, ski helmet use of cases and controls was noted on the questionnaire.

### 2.3. Statistical Analysis

All statistical analyses were performed using SPSS version 25 (IBM, New York, NY, USA). For the secondary aim, associated factors with the dependent variable risk-taking behavior (0: more risky, 1: more cautious) were analyzed. Simple binary logistic regression analyses were used to generate unadjusted odds ratio including 95% confidence intervals. Odds ratios including 95% confidence intervals of continuous variables were calculated for a 1-standard-deviation change of the total sample. 

For the first step of the primary aim, simple logistic regression analyses were performed for ACL injury as the dependent variable (0: uninjured, 1: ACL injured) to generate unadjusted odds ratio including 95% confidence intervals. In a second step, adjusted estimates of odds ratios and 95% confidence intervals were generated including all variables with a *p*-value < 0.20 of the simple regression analyses into a multiple binary logistic regression with ACL injury as the dependent variable. The *p*-value of < 0.20 was selected according to previous literature in the field [8,15]. *P*-values of less than 0.05 were considered statistically significant (two-tailed). Unless otherwise stated, data are presented as relative (absolute) frequencies and mean (standard deviation, SD).

## 3. Results

Out of the 528 skiers who participated in the study, 15.5% (82) suffered from an ACL injury and 84.5% (446) were uninjured. Mean age was 37.3 (SD: 11.9) years, mean BMI was 24.8 (SD: 3.5) kg/m^2^ and 50.9% (269) were female. Ski helmet use was 95.5% (504) of all skiers. A more risky behavior during skiing was reported by 26.9% (142) participants. In total, 412 skiers (78.0%) reported to be more skilled. Perceived skiing speed was fast/very fast, medium, and slow/very slow in 240 (45.5%), 244 (46.2%), and 44 (8.3%) skiers, respectively.

### 3.1. Factors Associated with Risk-Taking Behavior

Table 1 shows the results of the simple regression analyses regarding factors associated with self-reported risk-taking behavior. Sex, skill level and skiing speed emerged as significant. More risky skiers showed a higher rate of male participants and reported a higher skill level compared to more cautious skiers. In addition, a higher proportion of more risky skiers reported to ski with a (very) fast skiing speed compared to more cautious skiers. Median age and helmet usage rate was similar between more risky and more cautious skiers.

### 3.2. Factors Associated with ACL Injury

The results of the simple binary logistic regression analyses are displayed in Table 2. Age, risk-taking behavior, skill level, and skiing speed emerged as significant. ACL injured skiers reported a higher age, and a lower skill level compared to non-injured skiers. Sex distribution was similar between ACL injured and non-injured skiers. ALC-injured skiers reported significantly more often to be riskier and to ski more often with slow/or very slow speed compared to non-injured skiers. Helmet usage rate was similar between ACL injured and non-injured skiers.

Multiple binary regression analysis revealed the following factors to be significantly associated with ACL injury (Table 3): ACL injured skiers showed a higher age and a higher percentage of less skill skiers. In addition, a self-reported more risky behavior on ski slopes was associated with significantly higher odds for an ALC injury. Helmet usage and speed did not emerge as significant factors in the multiple model.

## 4. Discussion

The primary aim of this study was to evaluate whether self-reported risk-taking and ski helmet use are associated with an ACL injury in recreational alpine skiing. Multiple regression analysis revealed that an older age, a riskier behavior, and lower skill level were found to be significant risk factors associated with ACL injury, while ski helmet use was not. With regard to the secondary aim, a more risky behavior was associated with male sex, higher skill level, and higher speed, but not with ski helmet use.

### 4.1. Factors Associated with Risk-Taking Behavior

About 27% of the total cohort in this study self-reported a more risky behavior on the ski slope. Additionally, other studies found that about one fourth to one third of participants rated themselves as risky skiers or snowboarders [7,8,12,13,15]. 

Results of the simple regression analyses revealed that a more risky behavior on ski slopes was associated with male sex, higher skill level and higher self-perceived speed, but not with ski helmet use. Well in accordance, Ruedl et al. [12] measuring speeds of uninjured recreational skiers with a radar speed gun found that a self-reported riskier behavior was associated with younger age, male sex, higher skill level, lower BMI, and with on average 8 km/h higher speed, but not with helmet use. In addition, in a study including injured skiers and snowboarders, self-reported riskier behavior was associated with younger age, male sex, and higher skill level, while ski helmet use did not significantly differ between more risky and more cautious subjects [15]. In contrast to the above-mentioned studies, age did not differ between more risky and more cautious skiers in the underlying study. However, in a study by Llewellyn and Sanchez [20] on risk-taking in rock climbing, age did not predict risk-taking in rock climbing as both young and old climbers seemed motivated to take risks. These authors argued that the positive association between age and risk-taking may reflect the positive association between age and experience [20].

### 4.2. Factors Associated with ACL Injury

ACL injured skiers were significantly older compared to uninjured controls. In accordance, a recent study by Posch et al. [16] found a significant age difference between ACL injured and uninjured skiers (43 versus 40 years). In addition, Burtscher et al. [21] reported that female skiers with knee injuries were older compared to female skiers with non-knee injuries. Ekeland et al. [22] found a higher prevalence of knee injuries for adults compared to children (30 versus 22%) and more adults than children needing ambulance transport and treatment by physician or hospital suggesting that injuries suffered by adults were more serious than those suffered by children. With regard to ACL injury, this observation may be at least partly due to the fact that the valgus-external rotation, the most common self-reported ACL injury mechanism in recreational skiing [17,23,24], occurred most frequently in injured skiers aged between 40–50 years [24]. In addition, aging is associated with a progressive loss of neuromuscular function by a reduction of muscle mass and muscle quality and with changes in the biology, healing capacity, and biomechanical function of tendons and ligaments [25,26].

ACL injuries in this study were significantly associated with a self-reported more risky behavior. These findings are in contrast to earlier studies, which revealed that risk-taking behavior seems not to be primarily associated with injuries on ski slopes [27,28]. Bouter et al. [27] found lower scores in thrill and adventure seeking (TAS, a subscale of the personality trait sensation seeking) in injured skiers compared to a control group of uninjured skiers. Goulet et al. [28] compared uninjured skiers randomly selected on ski slopes, injured skiers, and skiers observed on slopes while performing thrill-seeking maneuvers (risk-taking group) with regard to attitudes toward risk-taking behavior and risk-taking behavior. Interestingly, results showed that injured skiers did not take more risk, but were less skilled compared to uninjured skiers while skiers from the risk-taking group were the most skilled [28]. However, these studies used cohorts of skiers with different injuries and not with the same diagnosis (e.g., an ACL injury) and were conducted probably including skiers with so-called long and unshaped traditional skis. The introduction of the short and shaped carving skis at the beginning of the new millennium seemed to increase mean speed on ski slopes by about 10 km/h [29] and to alter ACL injury mechanisms reported in the literature [23]. In addition, we found that riskier skiers reported to ski significantly faster compared to cautious skiers, which is in accordance with previous studies [12,19]. Fast and narrow carving turns can also result in an ACL injury without any fall [30].

In the present study, a lower skill level showed a higher odds ratio for an ACL injury than a riskier behavior (OR 6.7 versus OR 5.4). This seems to be in accordance with the findings by Bouter et al. [27] and Goulet et al. [28], who concluded that ski injuries are more likely due to a lower skill level than to higher risk-taking behavior. As Bouter et al. [27] found that injured skiers had lower TAS scores compared to uninjured skiers; they argued that skiers with higher TAS are better at handling the risk of several forms of physical exercise. Thus, they might be less prone to accidents and injuries compared to those with a relatively low TAS score who may be less skilled in estimating and handling the risk of recreational skiing [27]. In general, overall injury risk in alpine skiing seems to be higher with a lower skill level [31].

Ski helmet use was not a significant predictor for an ACL injury. Ski helmet use has steadily increased worldwide in the past two decades and reaches in the Alps up to 90% in adults and 100% in children [32]. In contrast to helmet use, no increase neither in head injury rate [5,33] nor in the overall injury rate has been observed [33,34] which would have been expected if risk compensation would play an important role. Studies even reported a decrease of head injury prevalence as well as head injury risk with increased ski helmet wear [5,33], as well as a constant or even lower total injury risk on ski slopes over the past decades [33,34]. Thus, available data from the literature and findings of this study do not support the risk compensation theory in this special field of ACL injuries and a riskier behavior in some ski helmet wearers seems to be no argument against the protective effect of ski helmet use. 

A few limitations have to be considered. Firstly, due to the restriction of ACL injured skiers to one ski clinic, a possible selection bias of ACL injured skiers cannot be excluded. However, a major part of knee injuries occurring in the study area was treated in this ski clinic and there are no indications of any source of selection bias. Secondly, a single question was used to assess risk-taking behavior, which might lead to under-reporting or over-reporting of health risk behaviors affected by cognitive and situational factors, especially in younger people [35]. However, this question to assess risk-taking behavior (i.e., more risky versus more cautious) on ski slopes appears to have face validity [8] and seems to be a valid single item approach related to the sensation seeking total score [13]. Thirdly, more than 90% of ACL injured and uninjured skiers used helmets, which might have prevented to detect a potential association between helmet use and ACL injury. 

## 5. Conclusions

In conclusion, a self-reported more risky behavior turned out as an associated factor with ACL injury, while this seems not to be true for ski helmet use. Furthermore, the present findings indicate that helmet use per se is not associated with higher risk-taking on ski slopes and with ACL injury. No support for the risk compensation hypothesis was found with regard to ACL injuries. Therefore, we doubt that ski helmet use increases the risk for ACL injury and recommend wearing a ski helmet due to reported protective effects. 

## Figures and Tables

**Table 1:** Simple regression analysis regarding factors associated with the dependent variable risk-taking behavior (0: more risky, 1: more cautious) on ski slopes.

	Risky Skiers (*n* = 142)		Cautious Skiers (*n* = 386)					
	Med	(IQR)	Med	(IQR)	OR	OR 95% CI lb	OR 95% CI ub	*p*-Value
Age, years ^a^	34.0	(26.0–43.0)	34.5	(29.0–45.0)	0.88	0.73	1.08	0.219
	**%**	**(n)**	**%**	**(n)**	**OR**	**OR 95% CI lb**	**OR 95% CI ub**	***p*-Value**
Sex								
male	63%	(90)	44%	(169)				
female	37%	(52)	56%	(217)	0.45	0.30	0.67	<0.001
Helmet use								
yes	96%	(137)	95%	(367)				
no	4%	(5)	5%	(19)	0.70	0.26	1.92	0.495
Skill level								
more skilled	94%	(134)	72%	(278)				
less skilled	6%	(8)	28%	(108)	0.15	0.07	0.32	<0.001
Speed								
fast/very fast	65%	(92)	38%	(148)	1 (ref)			
medium	34%	(48)	51%	(196)	0.39	0.26	0.59	<0.001
slow/very slow	1%	(2)	11%	(42)	0.08	0.02	0.32	<0.001

^a^ missing cases: *n* = 3, ACL: anterior cruciate ligament, Med: Median, IQR: Interquartile range, OR: unadjusted odds ratio, OR 95% CI: 95% confidence interval of the odds ratio, lb: lower bound, ub: upper bound, bold values represent significant factors.

**Table 2:** Simple regression analysis regarding factors associated with the dependent variable ACL injury (0: uninjured, 1: ACL injured) among recreational skiers.

	Uninjured Persons (*n* = 446)		ACL Injured Persons (*n* = 82)			OR 95% CI lb	OR 95% CI ub	*p*-Value
	Med	(IQR)	Med	(IQR)	OR			
Age, years ^a^	33.0	(26.0–45.0)	43.5	(38.0–53.3)	2.14	1.67	2.75	<0.001
	**%**	**(n)**	**%**	**(n)**	**OR**	**OR 95% CI lb**	**OR 95% CI ub**	***p*-Value**
Sex								
male	50%	(222)	45%	(37)				
female	50%	(224)	55%	(45)	1.21	0.75	1.93	0.439
Risk-taking behavior								
more cautious	76%	(337)	60%	(49)				
more risky	24%	(109)	40%	(33)	**2.08**	1.27	3.40	**0.003**
Helmet use								
yes	96%	(428)	93%	(76)				
no	4%	(18)	7%	(6)	1.88	0.72	4.88	0.196
Skill level								
more skilled	83%	(368)	54%	(44)				
less skilled	17%	(78)	46%	(38)	**4.07**	2.48	6.71	**<0.001**
Speed								
fast/very fast	46%	(203)	45%	(37)	1 (ref)			
moderate	48%	(215)	35%	(29)	0.74	0.44	1.25	0.259
slow/very slow	6%	(28)	20%	(16)	**3.14**	1.55	6.36	**0.002**

^a^ missing cases: *n* = 3 each, ACL: anterior cruciate ligament, Med. Median, IQR: Interquartile range, OR: unadjusted odds ratio, OR 95% CI: 95% confidence interval of the odds ratio, lb: lower bound, ub: upper bound, bold values represent significant factors.

**Table 3:** Multiple binary regression analysis with ACL injury (0: uninjured, 1: ACL injured) as dependent variable.

b	Standard Error of b		OR	OR 95% CI lb	OR 95% CI ub	*p*-Value
Factors						
Age, years ^a^	0.08	(0.01)	**1.30**	1.20	1.42	**<0.001**
Helmet use: no	1.08	(0.61)	2.95	0.90	9.69	0.075
Risk-taking behavior: more risky	1.69	(0.34)	**5.42**	2.81	10.47	**<0.001**
Skill level: less skilled	1.91	(0.35)	**6.74**	3.42	13.27	**<0.001**
Speed						
fast/very fast				1 (ref)		
moderate	−0.41	(0.31)	0.66	0.36	1.22	0.186
slow/very slow	0.76	(0.47)	2.13	0.85	5.30	0.105
Constant	−10.60		(1.28)	0.00		**<0.001**

^a^ missing cases: *n* = 3, ACL: anterior cruciate ligament, b: unstandardized regression coefficient, OR: unadjusted odds ratio, OR 95% CI: 95% confidence interval of the odds ratio, lb: lower bound, ub: upper bound, bold values represent significant factors, Nagelkerkes R^2^: 32.4%.
